# Diffuse Venous Malformation of the Uterus in a Pregnant Woman with Klippel-Trénaunay Syndrome Diagnosed by DCE-MRI

**DOI:** 10.1155/2016/4328450

**Published:** 2016-02-24

**Authors:** Nana Yara, Hitoshi Masamoto, Yuko Iraha, Akihiko Wakayama, Yukiko Chinen, Hayase Nitta, Tadatsugu Kinjo, Yoichi Aoki

**Affiliations:** ^1^Department of Obstetrics and Gynecology, Graduate School of Medicine, University of the Ryukyus, 207 Uehara, Nishihara, Okinawa 903-0215, Japan; ^2^Department of Radiology, Graduate School of Medicine, University of the Ryukyus, 207 Uehara, Nishihara, Okinawa 903-0215, Japan

## Abstract

*Background.* We experienced a rare case of a pregnant woman with Klippel-Trénaunay syndrome complicated with diffuse venous malformation of the uterus. This is the first report on the usefulness of dynamic contrast-enhanced-MRI for the diagnosis of diffuse venous malformation of the uterus.* Case Presentation.* A 23-year-old woman presented with convulsions and talipes equinus position of both lower limbs at 11 weeks of gestation. At 27 weeks, ultrasonography demonstrated tubular echolucent spaces throughout the myometrium. Dynamic MRI at 37 weeks revealed that the myometrial lesion was enhanced slowly and showed homogeneous enhancement even on a 10 min delayed image. Taken together with unilateral foot hypertrophy, varices, and port-wine stain, the patient was diagnosed as having Klippel-Trénaunay syndrome complicated with diffuse venous malformation of the pregnant uterus. The patient underwent elective cesarean section because of severe dystonia. The lower uterine segment was thickened and heavy venous blood flow was observed at the incision. Histological diagnosis of the myometrial biopsy specimen was venous malformation.* Conclusions.* Both diffuse venous malformation and Klippel-Trénaunay syndrome during pregnancy can involve considerable complications, in particular, massive bleeding during labor. Women who suffer from this syndrome should be advised about the risk of complications of pregnancy.

## 1. Introduction

Klippel-Trénaunay syndrome (KTS) is a rare congenital malformation characterized by the triad of capillary malformations, atypical varicosities or venous malformations, and bony or soft tissue hypertrophy most commonly affecting unilateral lower limbs. Klippel and Trénaunay were the first to recognize the vascular anomalies as the common cause for the defects in the skin, limbs, and organs [1]. Later, Parkes-Weber described arteriovenous fistula formation in the hypertrophic limbs of these patients [2]. These two diseases must be differentiated because they do not share etiology or treatment [3]. However, there is often confusion between the two, and the term Klippel-Trénaunay-Weber syndrome has been used. The morbidity of the disease is primarily related to vascular abnormalities, which can result in venous insufficiency, thrombophlebitis, cellulitis, limb disparity, and thromboembolic disease. Pregnancy complicated with this syndrome is believed to be associated with exacerbation of these complications and a high risk of thromboembolism and hemorrhagic complications [4, 5].

Diffuse venous malformation of the uterus accompanying pregnancy is an extremely rare condition and usually results in profuse bleeding on delivery as the dilated, thin-walled vessels fail to contract, which is even more severe during cesarean section because of incision of the involved uterine wall [6]. Careful management is mandatory to achieve successful pregnancy. We experienced a rare case of KTS complicated with diffuse venous malformation of the pregnant uterus.

## 2. Case Presentation

### 2.1. Case

**Figure 1:**
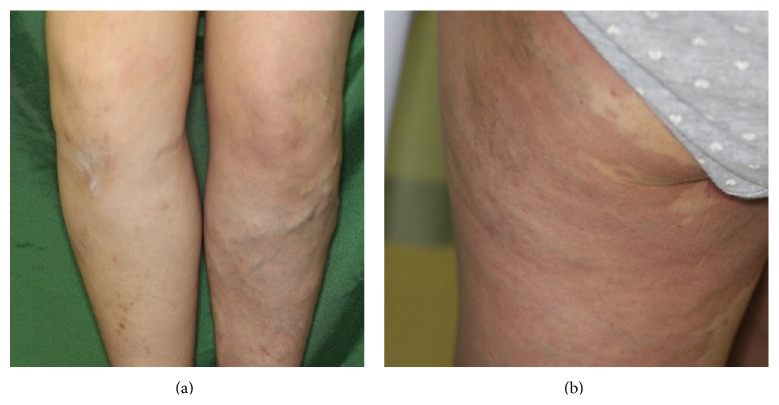
The patient had varices (a) and a port-wine stain (capillary malformation) on the left leg (b).

A 23-year-old, gravida 0 para 0 woman presented with focal left-sided convulsions, talipes equinus position of both lower limbs, and gait disturbance at 11 weeks of gestation. She was diagnosed with limb dystonia and referred to our hospital. She had varices and a port-wine stain (capillary malformation) on the left leg (Figure 1), and at the age of 15, she had undergone tibial epiphyseal line suppression surgery because of right foot hypertrophy. In the first trimester of this pregnancy, ultrasonography including Doppler flow measurements of the uterus and the pelvic vessels did not show any abnormality.

**Figure 2:**
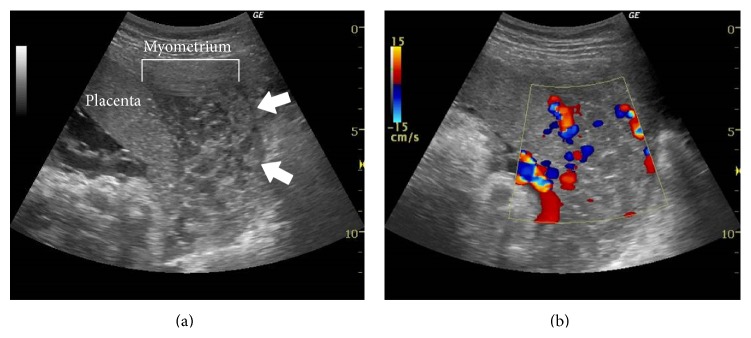
Ultrasonography demonstrated tubular echolucent spaces throughout the myometrium (a), and color Doppler showed blood flow within some of the cystic lesions (b) at 27 weeks of gestation.

**Figure 3:**
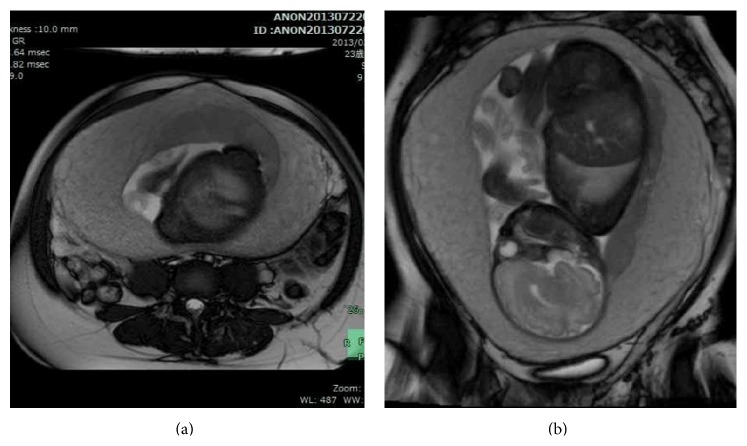
T2-weighted MRI showed a greatly enlarged uterus and diffuse myometrial thickening with a visible junctional zone between the endometrium and large high-intensity myometrium at 30 weeks of gestation ((a) transverse plane and (b) coronal plane).

**Figure 4:**
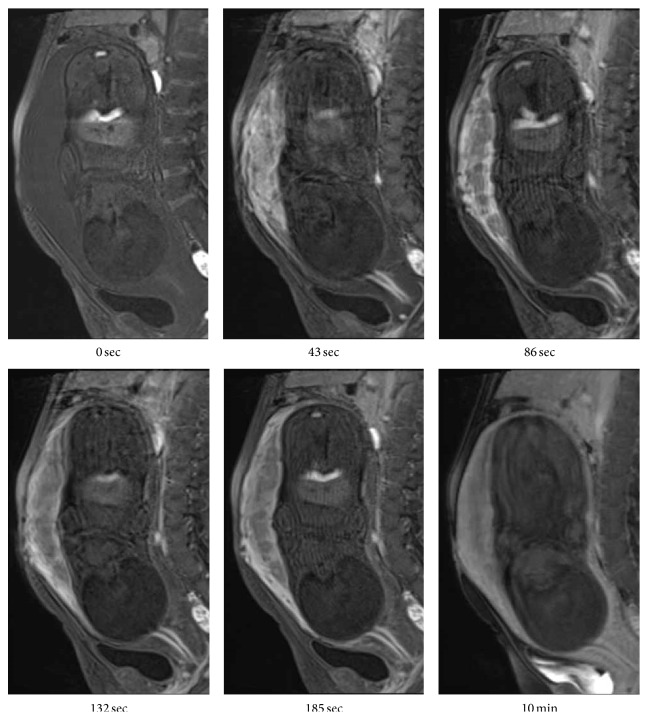
Dynamic contrast-enhanced-MRI using gadolinium at 37 weeks of gestation revealed that the myometrial lesion was enhanced slowly and showed homogeneous enhancement even on a 10 min delayed image, whereas the placenta demonstrated marked arterial enhancement and subsequent rapid washout.

At 27 weeks of gestation, ultrasonography demonstrated tubular echolucent spaces throughout the myometrium (Figure 2(a)), and color Doppler showed blood flow within some of the cystic lesions (Figure 2(b)). T2-weighted magnetic resonance imaging (MRI) showed a greatly enlarged uterus and diffuse myometrial thickening with a visible junctional zone between the endometrium and large high-intensity myometrium at 30 weeks of gestation, (Figures 3(a) and 3(b)). The border with the surrounding peritoneum was distinct, and there was no suggestion of extrauterine abnormalities. Because we thought that it had a considerable advantage to evaluate the blood flow of the lesion for accurate perinatal risk assessment, dynamic contrast-enhanced- (DCE-) MRI using gadolinium with diethylenetriaminepentaacetate (Gd-DTPA) was performed at 37 weeks of gestation, just before cesarean section, to assess vascularity of the myometrial lesion. DCE-MRI revealed that the myometrial lesion was enhanced slowly and showed homogeneous enhancement even on a 10 min delayed image, whereas the placenta demonstrated marked arterial enhancement and subsequent rapid washout (Figure 4). These findings indicated the abundant slow bloodstream in the lesion, leading to a consideration of diffuse venous malformation. Taken together with unilateral foot hypertrophy, varices, and port-wine stain capillary malformation, the patient was diagnosed as having KTS complicated with diffuse venous malformation of the pregnant uterus. The hemoglobin, hematocrit, platelet count, fibrinogen, D-dimer, and FDP levels just before the delivery were 10.5 g/dL, 29.7%, 12.3 × 10^4^/*μ*L, 194 mg/dL, 14.2 *μ*g/mL, and 30 *μ*g/mL, respectively.

At 37 weeks of gestation, the patient underwent elective cesarean section under general anesthesia because of severe dystonia. There were no abnormal vessels in the central zone of the uterine corpus, but in the lateral aspects of the uterus, many dilated vessels were present. The lower uterine segment was thickened to approximately 5 cm, and heavy venous blood flow was observed at the incision. A biopsy sample was taken from the myometrial surgical margin and the incision of the lower uterine segment was closed. A normal female infant weighing 2,198 g was delivered with Apgar scores of 4 and 7. Total operating time was 70 minutes and estimated blood loss was 3,792 g. During the procedure, the uterus contracted and the fundus was located at the navel level. The patient received a blood transfusion with 6 units of red cell concentrate. Enoxaparin sodium was administered for 6 days after the surgery to prevent thrombosis. The patient was discharged on day 8 postpartum without any considerable complication. Multiple cystic lesions in the myometrium were not observed by ultrasonography on day 7 after the cesarean section.

**Figure 5:**
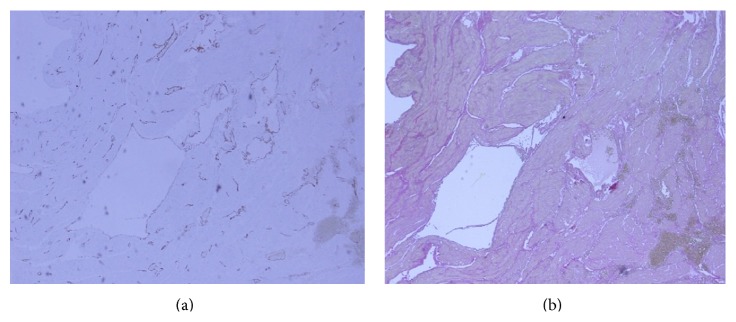
Histological examination showed numerous, variably sized, and thin-walled vessels distributed throughout the myometrium. The endothelial lining on the vessel wall was confirmed by strong immunoreactivity for CD31 and CD34, and lack of elastic fiber layer was observed by immunostaining with Elastica van Gieson.

Histological examination of the myometrial biopsy showed numerous, variably sized, and thin-walled vessels distributed throughout the myometrium. The endothelial lining on the vessel wall was confirmed by strong immunoreactivity for CD31 and CD34, and lack of elastic fiber layer was observed by immunostaining with Elastica van Gieson (Figure 5). The histological diagnosis was venous malformation.

## 3. Discussion

We reported a rare case of pregnant woman with KTS complicated with diffuse venous malformation of the uterus. The antenatal diagnosis for the diffuse venous malformation of the uterus may be difficult and requires a high index of suspicion by the radiologist as well as the obstetrician. Although the definitive diagnosis depends upon the histological examination of the uterus, diagnostic imaging of diffuse venous malformation demonstrates abundant abnormal blood vessels in the lesion. Ultrasonography showed that the entire uterine myometrium is thickened diffusely with numerous tubular echolucent spaces and comprised a large number of abnormal blood vessels. Blood flow in the lesion was slow; thus, color/power Doppler imaging may reveal slow or no flow in the affected area [7, 8]. In our patient, ultrasonography showed that the worm-like appearance of the echolucent areas was enlarged veins, probably in a venous malformation. The entire uterine wall had this appearance. Color Doppler images showed blood flow within some of the cystic lesions.

MRI revealed that the abundant slow bloodstream in the diffusely thickened myometrial layer was depicted as low intensity on T1-weighted image, and high intensity on T2 and contrast-enhanced images. Thanner et al. [8] reported the use of MRI for pregnancy-associated diffuse venous malformation of the uterus, demonstrating that the T1-weighted image with fat sat pulses after intravenous application of Gd-DPTA (0.1 mmol/kg) showed intensive enrichment of the contrast medium. Importantly, the DCE-MRI finding in our case, showing the slow enhancement of the myometrial lesion, and delay of the contrast medium in the lesion, allowed an accurate prenatal diagnosis. This is the first report on the usefulness of DCE-MRI for the diagnosis of diffuse venous malformation of the uterus.

Hysterectomy has been the most common treatment for diffuse uterine venous malformation to resolve the uncontrollable bleeding. Even prophylactic hysterectomy was performed in the past to prevent a rupture during pregnancy. However, it has been shown that, with conservative management and a vigilant approach, successful pregnancy can be achieved without resorting to a hysterectomy [7]. Lotgering et al. [9] demonstrated that marked autotransfusion from the hemangioma during labor contractions was clinically apparent and was confirmed by invasive hemodynamic monitoring. Furthermore, venous flow in the venous malformation as in the present case can be expected to cause bleeding that is usually controlled. During the cesarean delivery, our patient bled profusely from the venous malformations (estimated blood loss was 3,792 g) because of rupture of congested vessels or an inability of the uterus to contract sufficiently. The patient's condition was stabilized by infusion of oxytocin, packed red cells, and fresh frozen plasma. In addition, uterine artery embolization or ligation of the internal iliac artery could be alternative approaches, although no cases using these strategies have been reported. In pregnant women, this condition requires increased attention to maternal and fetal health to optimize the outcome of pregnancy. In some patients, the lesion completely disappeared by 6–12 months postpartum, as shown by imaging studies. However, it is unknown whether this regression was the result of the natural history of the lesion or a lack of hormonal stimulation in the postpartum period.

KTS was once thought to be a contraindication to pregnancy [5]. There are few case reports of pregnant woman with KTS complicated with diffuse venous malformation of the uterus. KTS presents difficult obstetric management issues, such as the risk of thromboembolism and hemorrhage, obstruction of the introitus, mandating cesarean delivery, and spinal hematoma at the site of the regional anesthesia administration, in which the normal physiologic changes during pregnancy seem to worsen the problems. Regarding fetal risks in these patients, the mode of inheritance is not well understood, and risk of fetal growth restriction may increase, according to some reports. With careful management, however, successful pregnancies can be achieved [5]. The arteriovenous malformations occurring in Klippel-Trénaunay-Weber syndrome may further complicate the pregnancy. Verheijen et al. [4] summarized five cases and concluded that the main risk was disseminated intravascular coagulation at or after delivery, and Richards and Cruz [10] observed diffuse venous malformation in the syndrome at 10 weeks of gestation during a pregnancy that was terminated for fear of life-threatening complications.

In conclusion, we experienced a rare case of a pregnant woman with KTS complicated with diffuse venous malformation of the uterus. Both diffuse venous malformation and Klippel-Trénaunay syndrome during pregnancy can involve considerable complications, in particular, massive bleeding during labor. Strict management and care should be offered. Women who suffer from this syndrome should be advised about the risk of complications of pregnancy.
